# Type 2 Diabetes Incidence and Mortality: Associations with Physical Activity, Fitness, Weight Loss, and Weight Cycling

**DOI:** 10.31083/j.rcm2311364

**Published:** 2022-10-25

**Authors:** Glenn A. Gaesser

**Affiliations:** ^1^College of Health Solutions, Arizona State University, Phoenix, AZ 85004, USA

**Keywords:** obesity, metabolic syndrome, cardiorespiratory, cardiovascular disease, exercise, weight fluctuation, body weight variability

## Abstract

Cardiometabolic diseases, including cardiovascular disease (CVD) and type 2 diabetes (T2D), are the leading cause of death globally. Because T2D and obesity are strongly associated, weight loss is the cornerstone of treatment. However, weight loss is rarely sustained, which may lead to weight cycling, which is associated with increased mortality risk in patients with T2D. Meta-analyses show that weight loss is not generally associated with reduced mortality risk in T2D, whereas weight cycling is associated with increased all-cause and CVD mortality. This may be attributable in part to increased variability in CVD risk factors that often accompany weight cycling, which studies show is consistently associated with adverse CVD outcomes in patients with T2D. The inconsistent associations between weight loss and mortality risk in T2D, and consistent findings of elevated mortality risk associated with weight cycling, present a conundrum for a weight-loss focused T2D prevention and treatment strategy. This is further complicated by the findings that among patients with T2D, mortality risk is lowest in the body mass index (BMI) range of ~25–35 kg/$m^{2}$. Because this “obesity paradox” has been consistently demonstrated in 7 meta-analyses, the lower mortality risk for individuals with T2D in this BMI range may not be all that paradoxical. Physical activity (PA), cardiorespiratory fitness (CRF), and muscular fitness (MF) are all associated with reduced risk of T2D, and lower risk of CVD and all-cause mortality in individuals with T2D. Reducing sedentary behavior, independent of PA status, also is strongly associated with reduced risk of T2D. Improvements in cardiometabolic risk factors with exercise training are comparable to those observed in weight loss interventions, and are largely independent of weight loss. To minimize risks associated with weight cycling, it may be prudent to adopt a weight-neutral approach for prevention and treatment of individuals with obesity and T2D by focusing on increasing PA and improving CRF and MF without a specific weight loss goal.

## 1. Introduction

Cardiometabolic diseases, including cardiovascular disease (CVD) and type 2 diabetes (T2D), are the leading cause of death globally [1]. An estimated 463 million people have T2D, and this number is projected to increase by 25% in 2030 and by 51% in 2045 [2]. Obesity is associated with increased risk of CVD and T2D, although lifestyle factors, such as physical activity and diet, are considered important to reduce risk of CVD and T2D [3, 4, 5, 6]. Because T2D and obesity are strongly associated, weight loss is the cornerstone of treatment [7]. However, weight loss is rarely sustained [8, 9, 10]. During the past 4 decades, worldwide obesity prevalence has doubled in 70 countries [11], and has tripled in the United States [12]. During this same period of time, the prevalence of weight loss attempts has increased substantially, and it is estimated that ~40%–50% of US adults attempt weight loss annually [13, 14]. Data from 2013–2016 National Health and Nutrition Examination Surveys indicated that two-thirds of adults with obesity tried to lose weight within the preceding year [13]. Thus, it could be argued that weight loss strategies have been largely unsuccessful at reducing obesity prevalence.

Due to the transient success of weight loss attempts, weight cycling is common, and is associated with health risks [14, 15, 16]. For example, the magnitude of the mortality risk associated with weight cycling [17, 18, 19] is comparable to, or greater than, that reported for obesity [20, 21, 22]. An important question is whether the risks of weight cycling outweigh the risks associated with obesity. This is especially relevant to individuals with T2D and obesity because weight loss is routinely advocated as a treatment strategy. Although obesity greatly increases the risk of T2D [23], the benefits of weight loss need to be considered in the context of potential risks associated with repeated episodes of weight regain that could lead to chronic weight cycling. Of additional consideration is the well documented finding of an obesity paradox in patients with T2D, which consistently shows that the body mass index (BMI, kg/$m^{2}$) associated with lowest mortality is in the BMI categories defined as overweight or moderately obese (i.e., BMI ~25–35 kg/$m^{2}$) [24, 25, 26, 27, 28, 29, 30]. Accordingly, it has been suggested that weight loss strategies that minimize weight cycling should be advocated [31, 32]. Given the poor success of weight loss attempts over the past several decades, such an approach may be impracticable.

Physical activity (PA), cardiorespiratory fitness (CRF), and muscular fitness (MF) also influence the risk associated with obesity and T2D [33, 34, 35, 36, 37, 38, 39, 40, 41]. PA is defined as any bodily movement produced by skeletal muscles that results in energy expenditure, and can categorized into occupational, sports, conditioning, leisure-time, or household activities [42]. CRF is a measure of maximal aerobic capacity and is usually assessed by an exercise test to volitional exhaustion [43]. MF generally reflects the integrated status of muscular strength and endurance [42], but in epidemiological studies is most frequently defined by assessing maximum force generation while performing a specific task (e.g., leg press, chest press, handgrip strength). Exercise training has well documented health benefits independent of weight loss [4, 5, 6], which is notable because exercise training alone rarely results in appreciable weight loss [44, 45, 46, 47]. This has implications for treatment because published data on the association between intentional weight loss and mortality risk in patients with T2D is sparse and inconsistent [48].

One of the objectives of this review was to examine the published data on mortality and CVD morbidity risks associated with weight loss and weight cycling in individuals with T2D. Another was to review published data on T2D incidence associated with PA, CRF, and MF, and the mortality risks associated with PA and CRF in persons with T2D. Comparison of associated risks may help to inform decisions about treatment strategies for individuals with T2D. This review relied primarily on data from prospective cohort studies and, when possible, meta-analyses of cohort studies.

## 2. Weight Loss and Mortality Risk in T2D

Although weight loss is routinely advocated for treating individuals with obesity, especially with comorbidities such as CVD and T2D, intentional weight loss is not consistently associated with reduced mortality risk [4]. This is particularly true for individuals with T2D. A meta-analysis of 3 observational cohort studies indicated that intentional weight loss in overweight or obese individuals with T2D was not associated with significantly reduced all-cause mortality risk (relative risk, RR = 0.90; 95% confidence interval, CI, 0.67–1.22) [48]. It is important to note that this meta-analysis exhibited considerable heterogeneity in risk estimates across the 3 cohort studies.

**Fig. 1.**
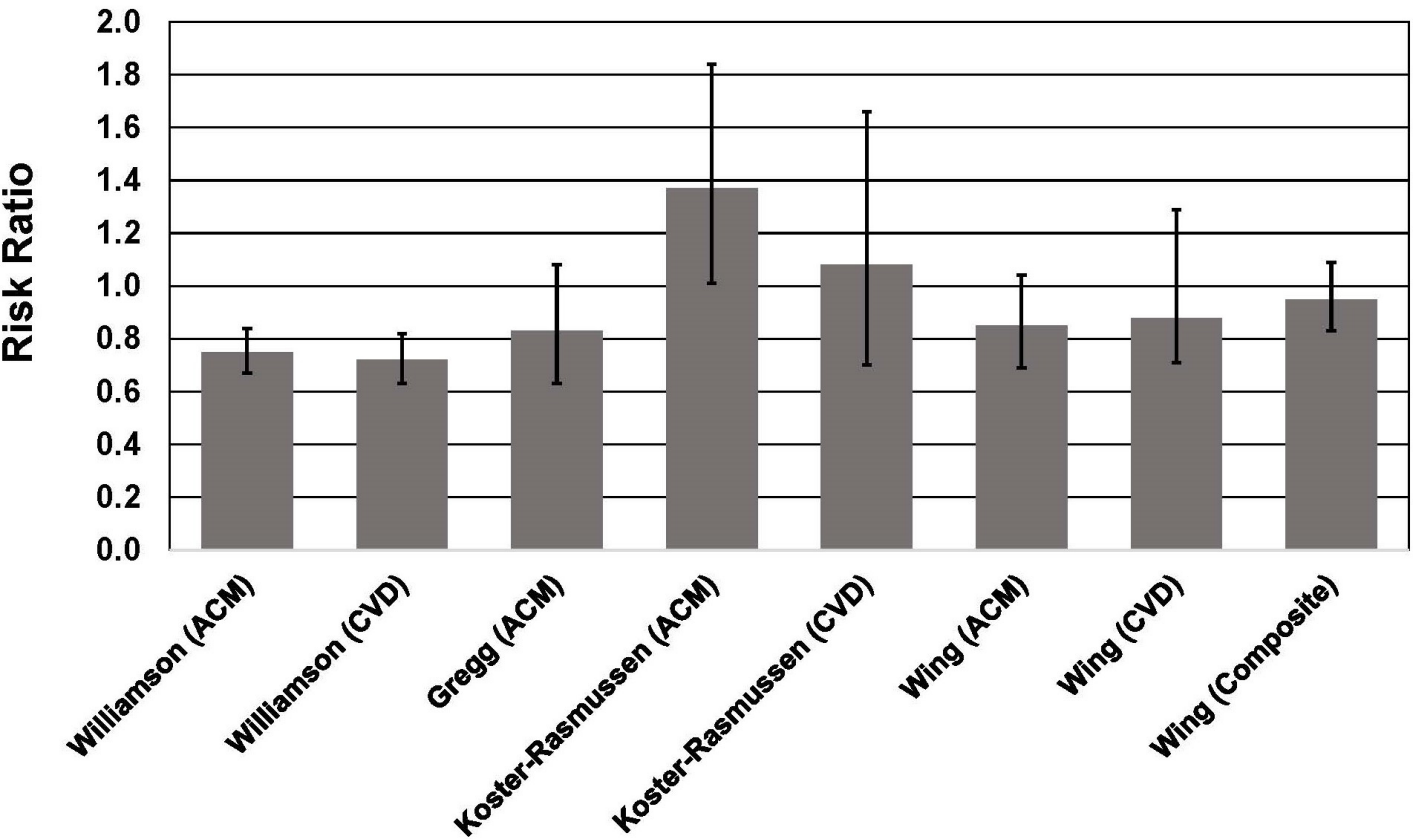
**Association between intentional weight loss and mortality risk in individuals with type 2 diabetes**. See text for description of studies. Vertical bars = 95% confidence intervals. ACM, All-cause mortality; CVD, cardiovascular disease mortality. In the study by Wing *et al*. [50], composite = death from cardiovascular causes, nonfatal myocardial infarction, nonfatal stroke, or hospitalization for angina.

For example, in the American Cancer Society’s Cancer Prevention Study I [49], self-reported intentional weight loss among men and women with T2D and BMI >27 kg/$m^{2}$ was associated with a 25% lower risk of all-cause mortality (RR = 0.75, 95% CI, 0.67–0.83) and 28% lower risk of CVD mortality (RR = 0.72, 95% CI, 0.63–0.82) (Fig. 1, Ref. [50]). Data on individuals with T2D and BMI >25 kg/$m^{2}$ in the National Health Interview Survey [51] showed that self-reported intentional weight loss was not associated with lower risk of mortality during 9 years of follow-up (Hazard Ratio, HR = 0.83, 95% CI, 0.63–1.08). Interestingly, just “trying to lose weight” but failing to do so was associated with a 23% lower risk of mortality (HR = 0.72, 95% CI, 0.55–0.96) (Fig. 1). These results suggest that the behaviors (e.g., exercise and diet) may be more important for reducing mortality risk than weight loss *per se*.

By contrast, among patients in the intervention arm of the Diabetes Care in General Practice randomized clinical trial [52], intentional weight loss was generally associated with higher mortality risk (Fig. 1). This result was primarily driven by patients with T2D and BMI >30 kg/$m^{2}$. During 13 years of follow-up, those with BMI >30 kg/$m^{2}$ in the “intention to lose weight group” had a 37% higher all-cause mortality risk (HR = 1.37, 95% CI, 1.01–1.84) for each 1-kg weight loss per year during a 6-year monitoring period. For patients with BMI <30 kg/$m^{2}$, intentional weight loss was not associated with mortality risk. In contrast to studies in which weight loss intention and amount were self-reported [49, 51], subjects in this study were weighed regularly during the physician-supervised 6-year monitoring period, with a median of 13 weights recorded during the monitoring period. Lowest mortality was observed in patients who maintained their weight during the follow-up period.

The findings of the Diabetes Care in General Practice study are consistent with the results of the Look AHEAD trial that found no benefit of an intensive lifestyle intervention that resulted in significant weight loss in adults with T2D and obesity [50]. During a median follow-up of 9.6 years, patients receiving the intensive lifestyle intervention had a HR (0.95, 95% CI, 0.83–1.09) for the primary outcome (death from cardiovascular causes, nonfatal myocardial infarction, nonfatal stroke, or hospitalization for angina) that was not different from the control group receiving diabetes support and education, despite achieving significantly greater weight loss throughout the study and greater reductions in hemoglobin A1c (HbA1c) (Fig. 1).

## 3. Weight Cycling and Mortality Risk in T2D 

**Fig. 2.**
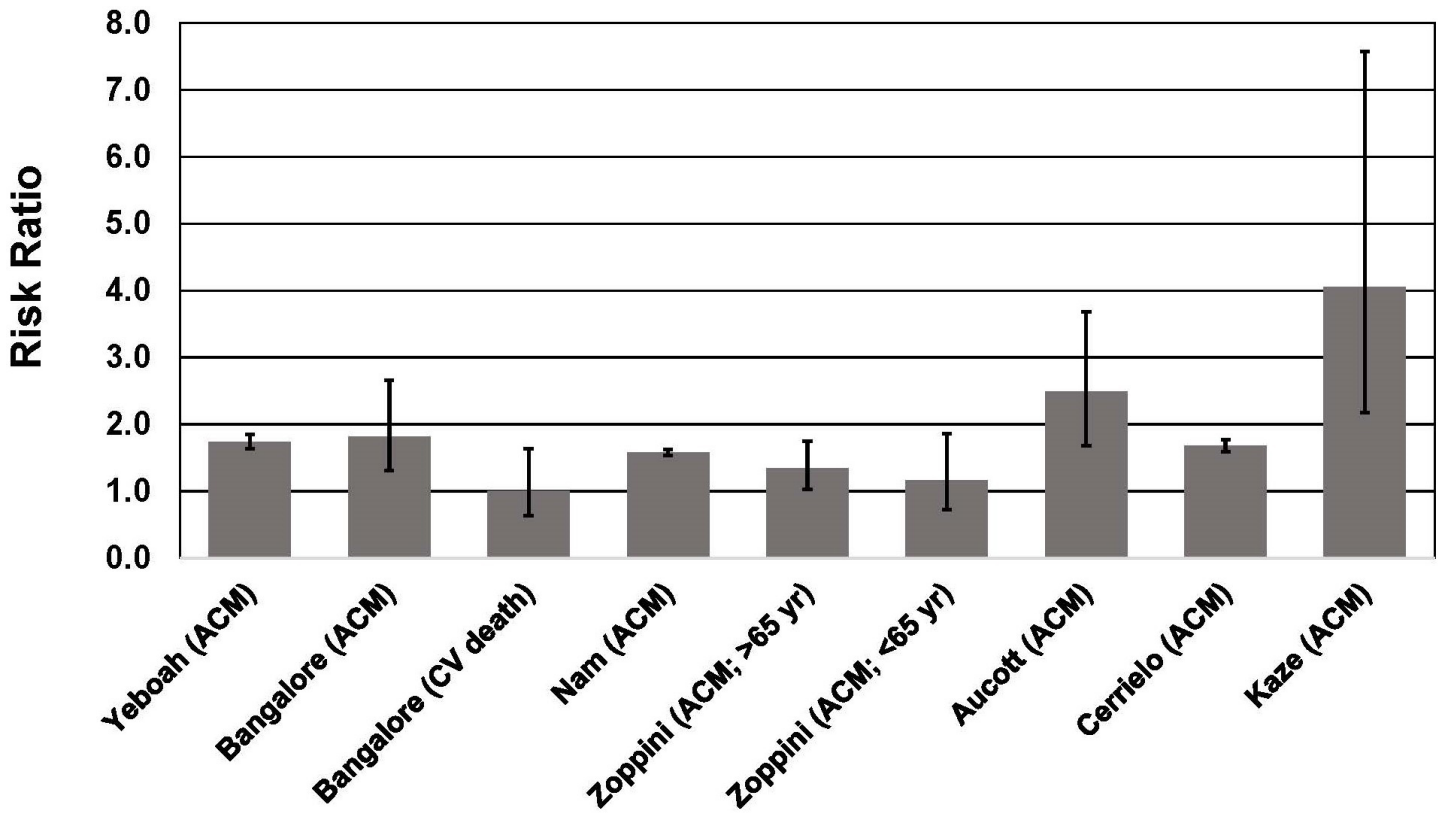
**Association between body weight fluctuation (weight cycling) and mortality risk in individuals with type 2 diabetes**. See text for description of studies. Vertical bars = 95% confidence intervals. ACM, All-cause mortality; CV, cardiovascular. In the study of Kaze *et al*. [55], a 15.28-fold higher risk of CVD mortality is not shown for purposes of maintaining scale on the y-axis.

**Fig. 3.**
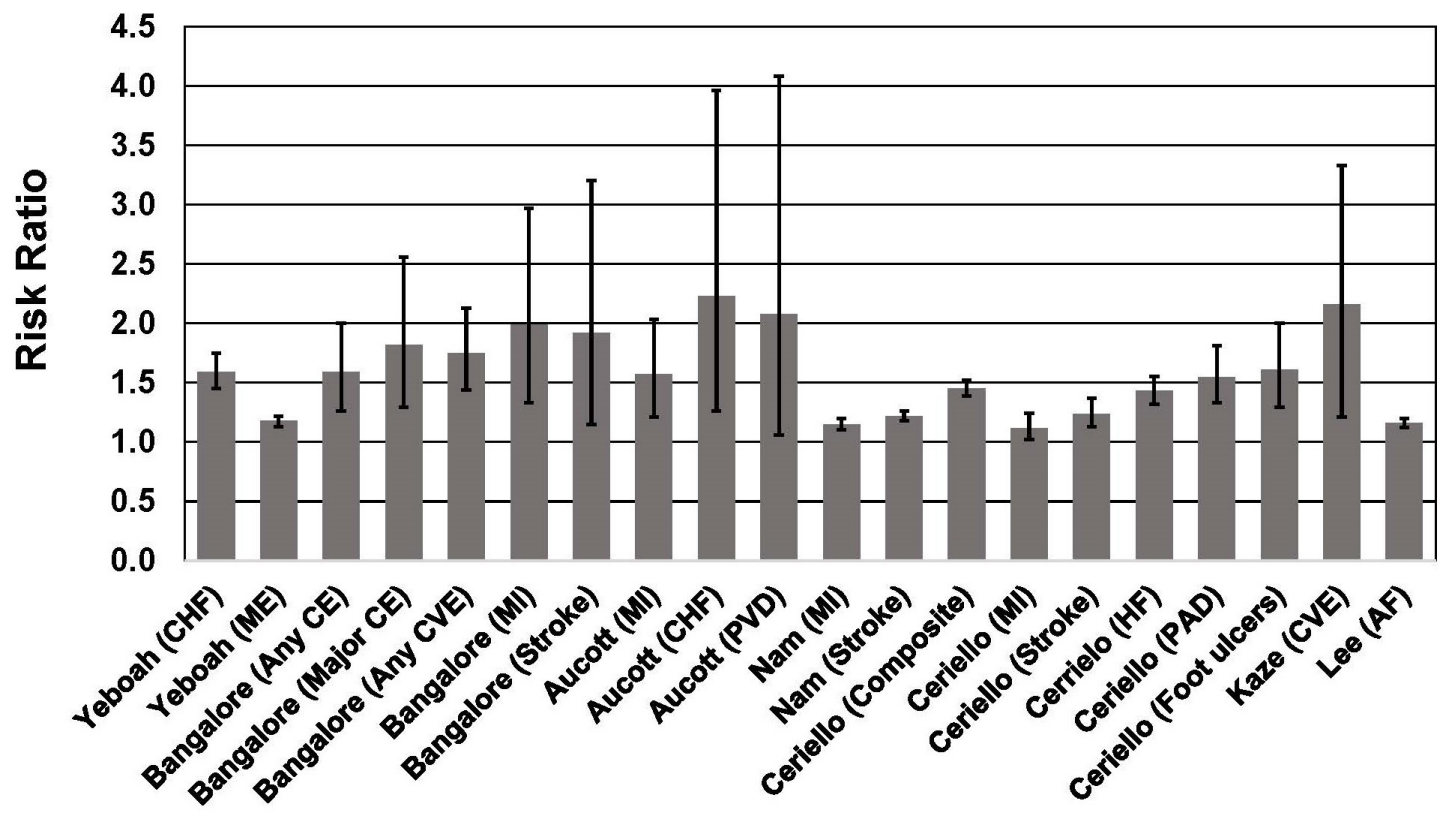
**Association between body weight fluctuation (weight cycling) and risk of adverse cardiovascular outcomes in individuals with type 2 diabetes**. See text for description of studies. Vertical bars = 95% confidence intervals. AF, atrial fibrillation; CE, coronary event; CHF, congestive heart failure; Composite, Nonfatal myocardial infarction, nonfatal stroke, and all-cause mortality; CVE, cardiovascular event; HF, heart failure; ME, microvascular events; MI, myocardial infarction; PAD, peripheral artery disease; PVD, peripheral vascular disease.

Several meta-analyses have shown that weight cycling is associated with a ~41%–53% higher risk of all-cause mortality [17, 18, 19] and a 36% higher risk of cardiovascular disease mortality [19]. The 36% higher risk of CVD mortality is consistent with a 35% higher risk of hypertension and 49% higher risk of CVD morbidity associated with weight cycling [19]. Higher morbidity and mortality risk associated with weight cycling has been well documented in T2D [53, 54, 55, 56, 57, 58, 59, 60, 61] (Fig. 2 Ref. [55], Fig. 3). A recent meta-analysis that included 6 studies of individuals with T2D, indicated that weight cycling was associated with higher risk of all-cause mortality [62]. Regardless of how weight cycling was defined (e.g., average successive variability of body weight; coefficient of variation of body weight; standard deviation of body weight), fluctuation in body weight was associated with a 50%–58% higher risk of all-cause mortality in patients with T2D. Results from these six studies [53, 55, 56, 58, 59, 61], and three additional studies [54, 57, 60], are described below and presented in Figs. 2,3.

In the ACCORD trial (Action to Control Cardiovascular Risk Factors in Diabetes), which included 10,251 participants with T2D, body weight variability during a mean 3.5 years of follow-up was associated with a 25% higher risk of the primary outcome (nonfatal myocardial infarction or stroke, or CVD death), 59% higher risk of heart failure, 74% higher risk all-cause mortality, and an 18% higher risk of microvascular events [56]. These associations were significant even after adjusting for BMI. The ACCORD trial is a multicenter factorial randomized controlled trial (RCT) designed to compare intensive blood pressure, glycemic, and lipid treatment with standard care in patients with T2D, ~91% with BMI >25 kg/$m^{2}$, and 62% with BMI >30 kg/$m^{2}$. The ACCORD trial was not a weight loss intervention; thus it is not known if weight variability was the result of intentional or unintentional weight loss. In this trial, higher BMI was also associated with higher risk of heart failure and all-cause mortality, but body weight variability appeared to be associated with greater risk. For example, compared with patients with BMI >30 kg/$m^{2}$ who were in the lowest quartile of body weight variability, patients with BMI <25 kg/$m^{2}$ at baseline but who were in the highest quartile of body weight variability had a 191% higher risk of CVD events (HR = 2.91, 95% CI, 1.35–6.28).

Among 6408 patients with T2D participating in one of three clinical trials of statins [53], weight cycling was associated with a significant increase of a composite endpoint that included coronary heart disease, death, myocardial infarction, resuscitated cardiac arrest, coronary revascularization, and unstable or new-onset angina. Subjects had a median of 12 body weight assessments during the interventions and follow-up periods of between 3.9 and 4.9 years. Body weight variability was calculated as the average absolute difference between successive body weight measurements. When expressed as a continuous variable, each 1 standard deviation (SD) increase in body weight variability was associated with an ~8%–21% higher risk of coronary and cardiovascular events, including mortality. Compared with patients in the lowest quintile of body weight variability, patients in the highest quintile of body weight variability had a 59% higher risk of any coronary event, 84% higher risk of major coronary event, 75% higher risk of any cardiovascular event, 82% higher risk of death, 99% higher risk of MI, and 92% higher risk of stroke. These associations were independent of traditional CVD risk factors, mean body weight, and weight change. Importantly, the associations between body weight variability and CVD risk increased with BMI. Consequently, the CV risks associated with weight cycling among patients with T2D were disproportionately observed in those with the highest BMI. Since weight cycling is more prevalent among individuals with high BMI [63, 64], it is plausible that higher risk associated with obesity may be in part due to adverse health effects of weight cycling.

Among 624,237 Korean adults with T2D, body weight variability over a 5-year period was associated with a 58% higher risk of all-cause mortality, a 22% higher risk of stroke, and a 15% higher risk of myocardial infarction during a follow-up period of 7–8 years [59]. The results were unchanged after adjustment for traditional CVD risk factors, and were observed in all age groups and both sexes. Higher mortality risk was observed in individuals with low and high BMI, although the HR was higher for BMI <25 kg/$m^{2}$ (53%) compared to BMI >25 kg/$m^{2}$ (36%). The higher mortality risk associated with weight variability was also significant (and similar) among individuals who experienced a weight loss of ≥5%, weight gain of ≥5%, or had a weight change of <5% during the 5-year period in which body weights were measured. These results indicate that body weight trajectory during the study did not affect adverse outcomes associated with weight cycling.

In a study of 1319 patients with T2D in the Verona Diabetes Study [60], variability in BMI during a 3-year period was associated with a 34% higher risk of mortality during a 10-year follow-up in adults ages >65 years, but was not associated with significantly higher mortality in adults <65 years (HR = 1.16, 95% CI, 0.72–1.86).

Among 29,316 adults with T2D in Scotland [58], weight variability based on multiple measurements over a 5-year period was associated with increased risk of all-cause mortality, myocardial infarction, and congestive heart failure during a mean follow-up of 5.2 years. Risk increased with higher degrees of weight variability. For example, the all-cause mortality HR for the subjects in the quartile with the highest coefficient of variation (CV) of body weight was 2.49 (95% CI, 1.68–3.68) compared to the quartile with the lowest CV of body weight. For congestive heart failure, quartiles 2–4 all had significantly increased risk (68%–123% higher) compared with the referent group with the lowest CV in body weight.

In the Swedish National Diabetes Register [61], which included 100,576 adults with T2D, increasing body weight variability over a 3-year period was associated with a 45% higher risk of the primary outcome (non-fatal MI or stroke; all-cause mortality) during a subsequent 5-year follow-up. For the highest quartile of body weight variability, all-cause mortality risk was 68% higher compared to the quartile with the lowest body weight variability.

The Look AHEAD trial reported that weight fluctuation was associated with increased mortality risk in the control group but not the intensive lifestyle intervention group [55]. In the control group, participants in the highest quartile of CV of BMI had a 4.06-fold greater risk of all-cause mortality, a 15.28-fold higher risk of CVD mortality, and a 2.16-fold higher risk of cardiovascular events. The highest quartile of CV for waist circumference (WC) was also significantly associated with a 1.84-fold higher risk of all-cause mortality and a 6.46-fold higher risk of CVD mortality. By contrast, CV for BMI and WC were not associated with higher risk for any outcome measure in the intervention group. The authors speculated that the lack of association in the intervention group could possibly be attributed to the exercise component of the intervention. Even partial weight regain after weight-loss interventions has been shown to result in reversal of cardiometabolic improvements that occurred during the weight-loss intervention [65], and exercise during a period of controlled weight regain can counter the adverse effects of weight regain on cardiometabolic risk markers [66, 67]. It is also worth noting that accumulation of visceral abdominal fat following liposuction can be entirely eliminated by exercise training [68]. Thus it is possible that the exercise component of the Look AHEAD intensive lifestyle intervention helped offset the deleterious impact of multiple episodes of weight regain following weight loss that could have occurred during the 6.7 years of follow-up.

The mechanisms underlying the higher risk associated with weight cycling are not well understood, although fluctuations in CVD risk factors, which could accompany repeated cycles of weight loss/weight gain, have been shown to be associated with higher mortality risk, especially CVD mortality [14, 31, 32]. Weight fluctuation is associated with increased risk of hyperinsulinemia and insulin resistance, elevated blood glucose and glycemic variability, dyslipidemia, and hypertension [14, 15, 31, 32, 69]. All of these could help explain the elevated risk of adverse CVD outcomes associated with weight cycling (Fig. 2).

In addition to the increased risk of mortality and adverse CVD outcomes associated with weight cycling, it is important to note that weight cycling is also associated with T2D incidence. Two meta-analyses indicated that weight cycling was associated with a 21% [19] and 33% [70] higher risk of T2D incidence. Additionally, among >6 million Korean adults, weight cycling was associated with a 26.3% higher risk of T2D and a 17.5% higher risk of hypertension [71]. Thus, in addition to increasing mortality risk among individuals with T2D, weight cycling may also increase risk of developing the disease.

A limitation of the studies on weight cycling is that weight loss intention was not assessed, and unintentional weight loss is more frequently associated with higher mortality risk compared with intentional weight loss [72]. However, given the high prevalence of weight loss attempts worldwide, and the fact that weight loss is routinely recommended for individuals with T2D and obesity, it is plausible that much of the weight cycling is a result of intentional weight loss attempts that inevitably lead to weight regain [8, 9, 10].

## 4. Obesity, T2D, and Mortality: Paradox, or Not?

The relationship between BMI and mortality is complex, and is influenced by fitness and physical activity [4, 37, 73]. An obesity paradox has been demonstrated for numerous chronic health conditions, in which lowest mortality is typically observed in individuals with BMI >25 kg/$m^{2}$. With no exceptions, seven meta-analyses have documented the existence of an obesity paradox in T2D, with lowest mortality risk associated with BMI in the range of ~25–35 kg/$m^{2}$ [24, 25, 26, 27, 28, 29, 30]. Reasons for the obesity paradox remain obscure, but it is worth noting that among healthy adults, some studies [74, 75, 76], including meta-analyses [21, 77, 78, 79], have shown that the lowest mortality risk in apparently healthy populations is in a similar BMI range as that associated with lowest mortality in adults with T2D. Thus, the relatively lower mortality risk associated with overweight and moderate obesity in T2D, as is consistently shown in meta-analyses, may not be all that paradoxical. Accordingly, Flegal and Ioannidis [80] recommended that the term “obesity paradox” be abandoned because it has no precise definition and that labeling counterintuitive findings a paradox adds no value.

It is also important to note that none of the cohort studies used in these meta-analyses included any measure of CRF, which is strongly inversely associated with mortality risk across all BMI strata in patients with T2D [33, 34, 35]. In the Henry Ford Exercise Testing Project (FIT Project), which included 8528 patients with T2D, after a 10-year mean follow-up, subjects with obesity had a 30% lower mortality rate compared to patients with BMI <25 kg/$m^{2}$. This was most evident among patients with the lowest CRF. Within each BMI group, there was a strong inverse association between CRF and mortality rate. Overall, compared to those in the lowest category of CRF (<6 METs), those with higher CRF (6–9.9 METs) had a ~50% lower mortality rate and those with the highest levels of CRF (>10 METs) had a 70% lower mortality rate. The lowest mortality rate was observed among individuals with BMI >30 kg/$m^{2}$ and high CRF (>10 METs). In this cohort, the lower mortality associated with CRF was independent of CVD risk factors, including hypertension, hyperlipidemia, diabetes, and smoking. In fact, an individual with a CRF >10 METs and ≥3 CVD risk factors had ~60% lower mortality rate compared to an individual with zero risk factors but a CRF <6 METs. These results highlight the importance of CRF, and especially of maintaining moderate-to-high CRF even in the presence of multiple CVD risk factors. The results also suggest that the absence of traditional CVD risk factors does not confer mortality benefit among individuals with T2D and low CRF.

The Look AHEAD trial is also instructive with regard to the associations between CRF, BMI, and mortality in adults with T2D and BMI >25 kg/$m^{2}$. In a secondary analysis of 4773 adults, ages 45–76 years, after a mean follow-up of 9.2 years, all-cause mortality rate was 30% lower per SD higher MET level (1 MET = 3.5 mL $O_{2}$/kg/min). Also, for each SD higher MET level, CVD mortality was reduced by 55%, and a composite CV outcome was reduced by 28% [81]. These reductions were evident with and without BMI in basic multivariate model. By contrast, when adjusted for CRF, BMI was not associated with all-cause or CVD mortality, or higher risk of MI, stroke, angina, or heart failure. In fact, BMI was protective from stroke, MI and composite CVD outcomes when adjusted for CRF.

## 5. Fitness, Physical Activity, Sedentary Behavior, and Prevention of T2D

**Fig. 4.**
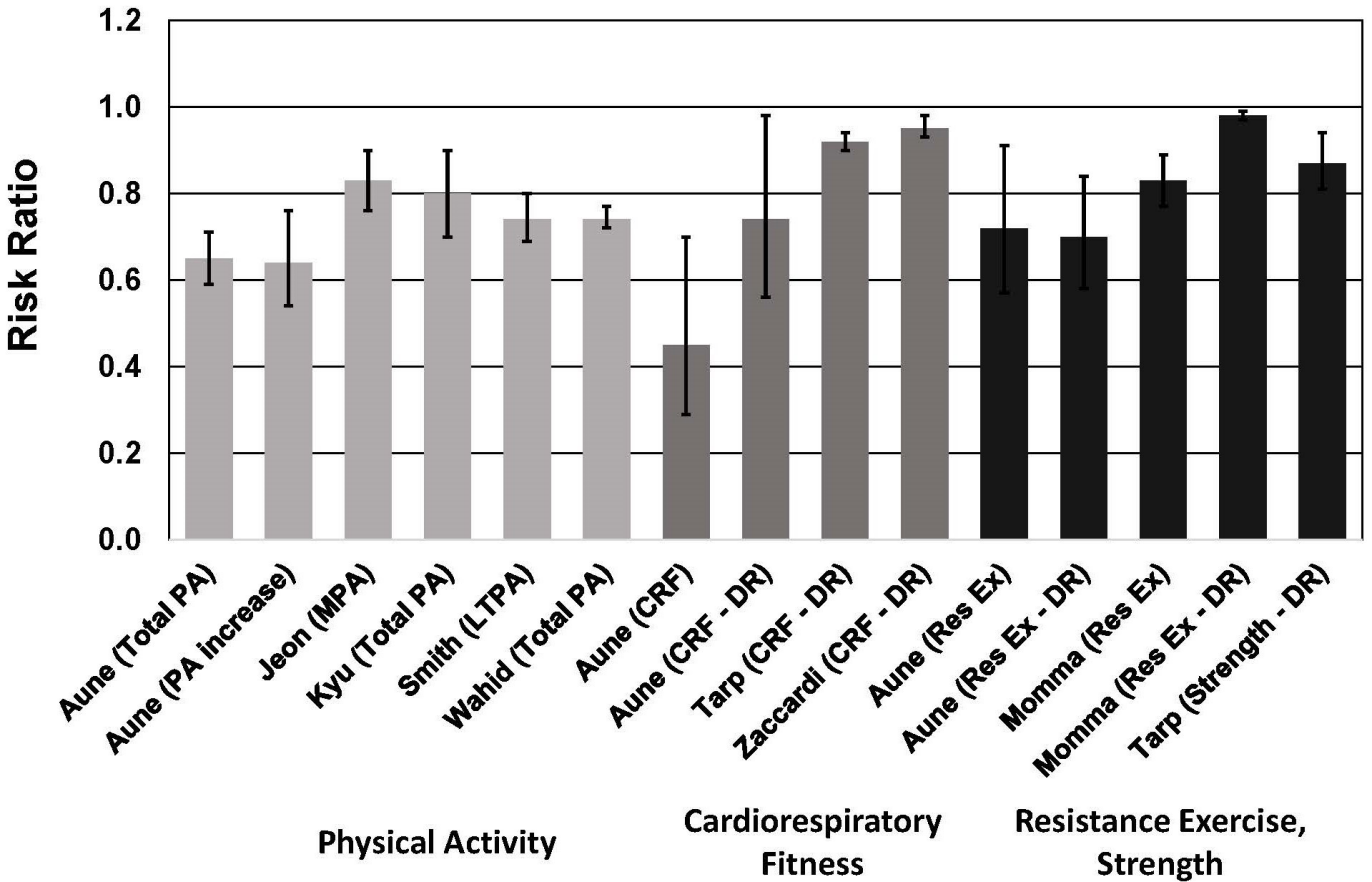
**Meta-analyses of cohort studies on the association between risk of type 2 diabetes and physical activity, cardiorespiratory fitness, resistance exercise and muscular strength**. See text for description of meta-analyses. Vertical bars = 95% confidence intervals. PA, physical activity; MPA, moderate-intensity physical activity; LTPA, leisure time physical activity; CRF, cardiorespiratory fitness; Res Ex, resistance exercise; DR, dose-response. In Aune *et al*. [82], DR = per 20 mL $O_{2}$/kg/min (~5.7 METs) for CRF and per 5 hours/week of resistance exercise; in Tarp *et al*. [39] and Zaccardi *et al*. [36], DR = per 1 MET (3.5 mL $O_{2}$/kg/min) higher cardiorespiratory fitness; in Momma *et al*. [89], DR = per 10 min/week of resistance exercise training; in Tarp *et al*. [39], DR = per 1 standard deviation in muscular strength.

Five meta-analyses have been published on the association between PA and risk of T2D. These meta-analyses consistently show lower risk associated with higher PA, with the highest PA levels corresponding to a 13% to 35% lower risk of T2D [82, 83, 84, 85, 86] (Fig. 4). These meta-analyses included data from 67 publications (not including duplications), with a total of ~2,160,445 men and women. Most of the cohorts were from the United States, China, Japan, South Korea, Australia, and 11 European countries. In two of the meta-analyses [82, 83], all of the included studies (59 of the 67 total across all five meta-analyses) were judged to have scores of 7–9 (high quality) on the 9-point Newcastle-Ottawa Scale (NOS) for quality assessment [87]. In one meta-analysis [84], 17 of 28 studies had NOS scores ≥7, and only 1 had a score <4. In the smallest meta-analysis [85], with only 3 studies, 2 had NOS scores ≥7 and 1 had a score of 4. One meta-analysis did not report whether the authors rated the quality of studies [86], but all of the studies included in this meta-analysis were also included in one or more of the other four meta-analyses. Thus, most of the studies included in these meta-analyses were assessed as high quality. Heterogeneity was only significant in one of the meta-analyses [84]. It is important to note that all of the 27 relative risks included in this meta-analysis were <1.0, with heterogeneity largely due to the large range in differences in relative risks (0.34 to 0.96). Significant publication bias was evident in two of the meta-analyses [82, 83].

Four of these meta-analyses demonstrated nonlinear dose-response associations, with steeper risk reductions associated with lower levels of PA [82, 83, 84, 85]. In two meta-analyses, PA equal to 11.25 MET-hour/week was associated with a 26% lower risk of T2D [84, 85]. This amount of PA corresponds to ~150 min/week of moderate-intensity PA (e.g., for a 4.5-MET activity such as walking at ~3.5 mph). These observations are important because the majority of adults with T2D do not meet the minimum PA guidelines [88].

The results of one meta-analysis highlighted the importance of increasing PA from a baseline sedentary level. For individuals who increased their PA from a low level to a moderate or high level, risk of T2D was reduced by 36% [82]. This was comparable to the 41% lower risk for individuals who had a consistently high level of moderate-to-vigorous PA (MVPA).

In addition to PA, CRF [36, 39, 82] and MF [39, 82, 89] are also associated with lower risk of T2D (Fig. 4). These meta-analyses included data from 17 publications for CRF and 15 publications for MF (not including duplications), with a total of ~1.6 million men and women in the CRF studies and ~1.9 million men and women in the MF studies. The majority of these participants consisted of ~1.5 million male military conscripts from Sweden [90], and were included in only one of the meta-analyses [39]. Cohorts from the United States, Canada, Finland, Sweden, Denmark, England, Switzerland, and Japan were included, as well as 17 countries in the Prospective Urban Rural Epidemiology (PURE) study. In two of the three meta-analyses on CRF [36, 82], all of the included studies (13 of the 17 total across all three meta-analyses) were assigned NOS scores of ≥7. In the other meta-analysis [39], 4 of 10 studies were assigned NOS scores >7 and the other 6 studies had NOS scores of 5 or 6. All three of the meta-analyses reported significant heterogeneity [36, 39, 82]. However, this was largely due to the wide range in relative risks reported (0.09 to 1.02), and it is important to note that all but 1 of the relative risks in these 17 studies were <1.0. Significant publication bias was evident in only one of the meta-analyses [82], and that was due to 1 study.

For CRF, each 1-MET (3.5 mL/kg/min) higher level of CRF was associated with a ~5%–8% lower risk of T2D [36, 39, 82]. The risk reduction estimates in these meta-analyses included adjustments for BMI. In support of these observations are results from the Coronary Artery Risk Development in Young Adults cohort, which indicated that a decrease in CRF over a 7-year period was associated with a 22% higher risk of T2D in women and a 45% higher risk of T2D in men during the 20 years of the study [91]. These findings highlight the importance of CRF for reducing risk of T2D, and are consistent with the finding that CRF is positively correlated with pancreatic beta cell function, independent of fatness in individuals with the metabolic syndrome [92].

For the meta-analyses on MF, one reported that all included studies had NOS scores ≥7 [82], one reported that 4 studies had NOS scores ≥7 and 6 studies had NOS scores between 4–6 [39], and one reported that all 5 studies included in the meta-analysis had NOS scores between 4–6 [89]. Only one of the meta-analyses reported significant heterogeneity [39], and none reported significant publication bias.

Three meta-analyses demonstrated that muscular strength was associated with a 13%–28% lower risk of T2D [39, 82, 89]. In categorical analyses, the highest strength group had a 17%–28% lower risk of T2D [82, 89]. In dose-response analyses, each 1-SD increase in muscular strength was associated with a 13% lower risk of T2D [39], and each 10 min/week of strengthening exercises was associated with a 2% lower risk of T2D [89]. In the latter dose-response study, risk decreased markedly until about 60 min/week of strengthening exercises.

On the opposite end of the PA continuum, it has also been shown that sedentary behavior, such as sitting time and watching television, increases risk of T2D [93, 94, 95, 96]. Although PA attenuates this relationship, sitting time and sedentary behavior are significantly associated with T2D risk independent of PA [93, 95]. A meta-analysis of 5 cohort studies indicated that, compared to the group with the lowest amount of sitting time, individuals with the highest amount of daily sitting time had a 13% higher risk of T2D [93]. Even when adjusting for PA, a high amount of daily sitting was associated with a 10% higher T2D risk. In another meta-analysis of 5 studies, the highest amount of total daily sedentary time was associated with a 91% higher risk of T2D [94]. Thus, reducing risk of T2D requires both increasing PA and fitness, as well as reducing time spent in sedentary activities.

It must be acknowledged that intensive lifestyle interventions accompanied by significant weight loss have also been documented to reduce T2D incidence [3]. However, when looking at the various intensive lifestyle interventions to prevent T2D, it is not apparent that significant weight loss is obligatory to achieve benefit. In a systematic review and meta-analysis of 7 large lifestyle interventions, T2D risk reduction across all interventions were similar, ranging between 48% and 57%, yet weight reduction among the studies varied considerably, with some of interventions showing minimal, if any, weight loss [3].

## 6. Increasing Physical Activity and Fitness to Reduce Mortality Risk in T2D

**Fig. 5.**
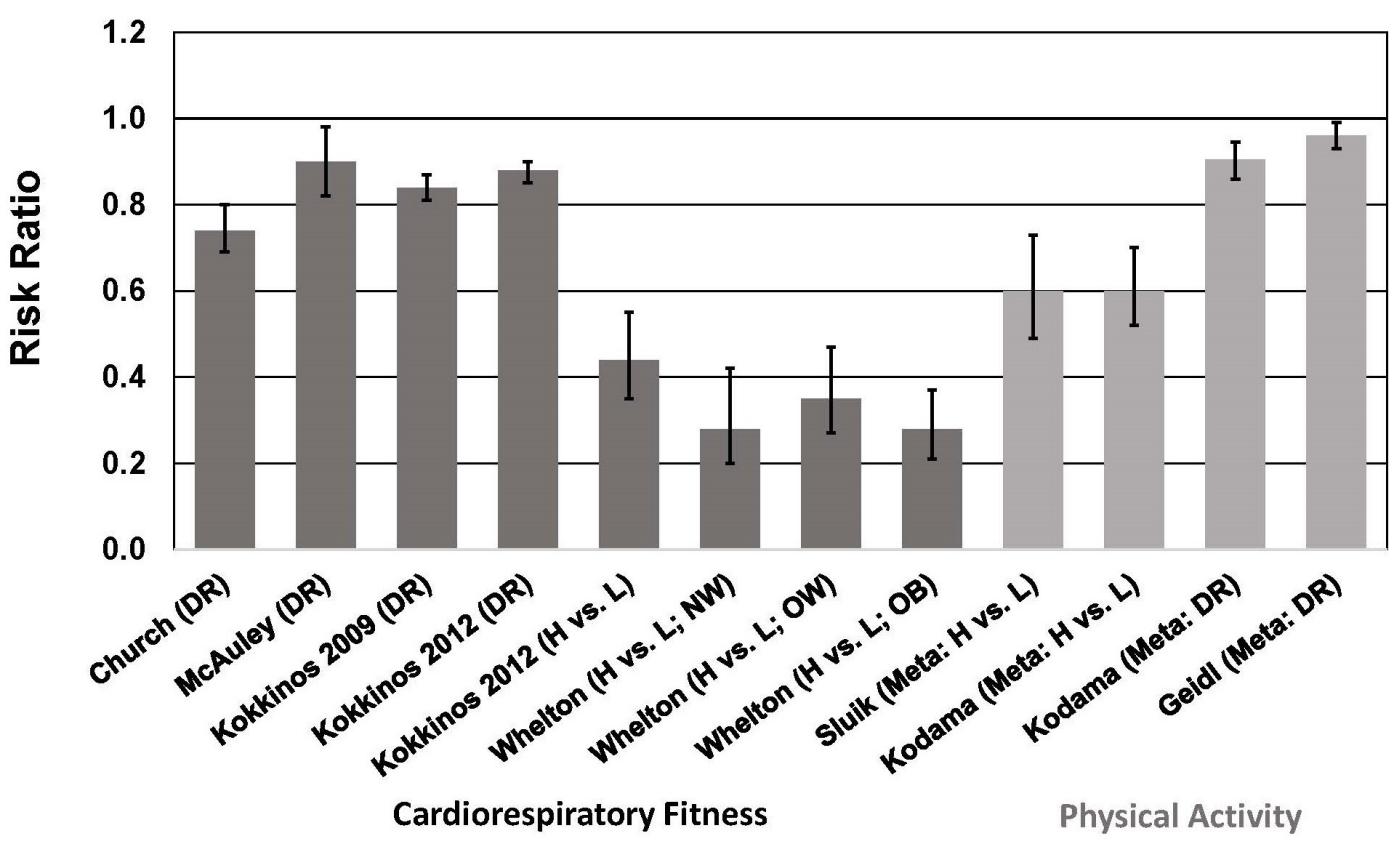
**Association between cardiorespiratory fitness (cohort studies) and physical activity (meta-analyses) and all-cause mortality risk in individuals with type 2 diabetes**. See text for description of studies. Vertical bars = 95% confidence intervals. ACM, all-cause mortality; DR, dose-response; H, highest category of CRF or PA; L, Lowest category of CRF or PA; Meta, meta-analysis; NW, normal weight, BMI = 18.5–<25 kg/$m^{2}$; OW, overweight, BMI = 25–<30 kg/$m^{2}$; OB, obese, BMI ≥30 kg/$m^{2}$. In all CRF studies, DR = per 1 MET (3.5 mL $O_{2}$/kg/min) higher CRF. In Kodama *et al*. [108], DR = per 1 MET-hour/day higher PA level; in Geidl *et al*. [107], DR = per 10 MET-hour/week higher PA level.

PA [97, 98, 99, 100], CRF [101, 102], and MF [103, 104, 105, 106] are all inversely associated with all-cause and CVD mortality, and these associations are independent of BMI. Among individuals with T2D, data from three meta-analyses demonstrate that higher PA is associated with lower all-cause mortality risk [107, 108, 109] (Fig. 5, Ref. [107, 108]). These meta-analyses included data from 20 publications (not including duplications), with a total of 47,467 men and women. The cohorts were from the United States, Japan, and 11 European countries. Only one of the meta-analyses assessed study quality with the NOS [109], in which 8 of the 12 included studies were assigned a score of ≥7, and 4 were assigned a score of 6. One meta-analysis did not report quality assessment [108], and one [107] used the GRADE (Grading of Recommendations, Assessment, Development and Evaluation) approach to assess certainty of evidence [110]. This meta-analysis of 6 studies graded the certainty of evidence as “low”. All three meta-analyses reported significant heterogeneity. Only one of the meta-analyses reported evidence of publication bias [108]. As discussed above, the significant heterogeneity was attributable to the large range of relative risks for lower mortality risk associated with higher CRF, with all but 1 of the studies reporting a relative risk <1.0.

In meta-analyses that included a total of 12 cohort studies of individuals with T2D, total PA, leisure-time PA, and walking were associated with a ~40% lower risk of all-cause and CVD mortality when comparing highest vs. lowest categories of PA [109] (Fig. 5). These risk reductions are similar to those reported in another meta-analysis showing a 40% lower risk of all-cause mortality (13 studies) when comparing highest vs. lowest categories of PA [108]. In a dose-response analysis, this study showed that each 1 MET-hour/day (~70–140 min/week of moderate-intensity PA, depending on MET level) increase in PA was associated with a 9.5% lower risk of all-cause mortality (Fig. 5). This meta-analysis also showed a 29% lower risk of CVD risk when comparing highest vs. lowest categories of PA, and a 7.9% lower risk of CVD per 1 MET-hour/day increase in PA in a dose-response analysis [108]. A more recent dose-response meta-analysis indicated that each 10 MET-hour/week (~100–200 min/week of moderate-intensity PA, depending on MET level) increase in PA was associated with a 4% lower risk of all-cause mortality [107]. The reason for the marked difference in risk reduction between the two dose-response meta-analyses is not apparent. The more recent meta-analysis included only 6 studies (3 of which were not included in the earlier meta-analysis), which makes comparisons difficult.

No meta-analyses have been published on the association between CRF and mortality in patients with T2D. However, several cohort studies have been published to confirm this association in T2D, and that the inverse association between CRF and mortality is independent of BMI [33, 34, 35, 40, 41]. Among men with T2D in the Aerobics Center Longitudinal Study [40], male veterans with T2D [33, 34, 35], and men and women in the Henry Ford Exercise Testing Project [41], higher CRF was associated with lower risk of all-cause mortality, and this association was observed in all BMI strata. Each 1-MET increase in CRF was associated with a 10%–18% survival benefit [33, 34, 35], which is comparable to the 11% lower all-cause mortality risk associated with a 1-MET higher CRF level reported in a recent meta-analysis of 37 cohort studies involving more than 2.2 million participants [111].

These data from cohort studies based on single point assessments of PA or CRF suggest that increasing PA and/or CRF would reduce mortality risk in individuals with T2D. Abundant evidence in healthy populations shows that increasing PA [112, 113, 114, 115, 116, 117, 118, 119] or improving CRF [120, 121, 122, 123, 124, 125, 126, 127] (determined by 2 or more assessments over time) is associated with reduced risk of all-cause and CVD mortality. Risk reductions for increasing PA are in the range of 15%–50% [4], whereas those associated with improvements in CRF are even greater. Moving from “low fit” to a higher fitness category is associated with a 30%–60% reduction in mortality risk [4], and in dose-response analyses, each 1-MET increase in CRF is associated with a 14%–29% reduction in all-cause mortality [4].

Unfortunately, there are limited data on individuals with T2D with regard to mortality risk reductions associated with either increasing PA or improving CRF. Among persons with T2D in the European Prospective Investigation into Cancer and Nutrition Study, changes in cycling behavior was associated with mortality risk [128]. Information on PA and cycling behavior was obtained at two time periods, 5 years apart. Compared with people who reported no cycling at baseline, during a mean follow-up of 7.7 years, those who took up cycling experienced a 35% lower risk of all-cause mortality and a 51% lower risk of CVD mortality. These risk reductions were independent of other PA. These results are consistent with previous reports showing that PA is associated with lower mortality rates in T2D [107, 108, 109]. These results on individuals with T2D are similar to those from a study of Danish adults which showed that taking up cycling was associated with a 22% lower risk of all-cause mortality during a follow-up of ~10–13 years [116]. It is worth noting that in this Danish population, cycling was not associated with weight loss or a reduction in the incidence of overweight or obesity during the follow-up [129].

The reduced mortality rate in persons with T2D who have high levels of PA and/or CRF could be due to many factors, including improved vascular endothelial function [130, 131, 132], reductions in visceral abdominal and ectopic fat [133, 134], and molecular adaptations in fat cells that improve “metabolic fitness” of adipose tissue [135, 136, 137]. These exercise-induced adaptations occur with little, if any, loss of total body fat [4]. Lower mortality risk in physically active persons with T2D may also be attributable in part to the beneficial effect of exercise on heart rate variability (HRV) [138]. Low HRV is associated with increased mortality risk [139], and HRV is reduced in patients with T2D [140]. A systematic review of 15 exercise intervention studies in patients with T2D showed that exercise training increased HRV [138]. Risk reduction may also be attributable to improvements in CVD risk factors, as described in the next section.

## 7. Exercise Training, Weight Loss, and CVD Risk Factors

Weight loss is associated with improvements in risk factors for CVD and T2D, as consistently documented in meta-analyses of RCTs [141, 142, 143, 144, 145, 146, 147, 148]. Weight loss in these studies was typically achieved by energy-restriction, either alone or in combination with exercise, and in one meta-analysis by medication [143]. Thus it is difficult to distinguish whether the improvements in risk factors are attributable to weight loss *per se*, or to changes in diet quality and/or exercise. As discussed below, improvements in CVD risk factors with exercise training are similar in magnitude to those reported in weight loss studies.

Weight loss interventions have been shown to reduce HbA1c by ~0.2% to 0.9% [141, 144, 147]. These reductions are similar to the reductions of ~0.2%–0.8% reported for exercise intervention studies [149, 150, 151, 152]. In the exercise RCTs, it is unlikely that weight loss accounted for the reductions in HbA1c because either weight loss did not occur with exercise training [149] or the magnitude of weight loss with exercise training was unrelated to the magnitude of reduction in HbA1c [152].

Increases in blood concentrations of high-density lipoprotein cholesterol with exercise training (2–5 mg/dL) [153, 154, 155, 156, 157] are comparable to those for weight loss interventions (1–4 mg/dL) [141, 142, 143, 144, 148], and reductions in low-density lipoprotein cholesterol for exercise training (3–10 mg/dL) [153, 155, 158] are similar to those reported for weight loss interventions (1–15 mg/dL) [141, 142, 143, 147, 148]. Reductions in blood triglyceride concentrations tend to be greater with weight loss interventions (11–58 mg/dL) [141, 147, 148] compared to exercise training (5–26 mg/dL) [153, 155, 157, 159].

Systolic and diastolic blood pressure reductions with exercise training (2–5 mmHg) [160, 161, 162, 163, 164, 165] are also similar to those reported in weight loss studies (1–5 mmHg) [141, 143, 144, 145, 146, 147]. Even though some weight loss may occur with exercise training, correlations between changes in blood pressures and body weight are <0.10 [166].

As mentioned above, these results must be interpreted with caution because the weight loss studies used diet and/or exercise in combination, or weight loss medications. Since diet alone and exercise alone can improve CVD risk factors in the absence of weight loss [4, 5], conclusions about the independent effects of weight loss must be considered in this context.

## 8. Summary and Conclusions

Prevalence of obesity and incidence of T2D have increased steadily during the past 40 years, and are projected to increase further in the next few decades. Although weight loss remains the cornerstone of treatment, empirical evidence strongly suggests that weight loss strategies have been largely ineffective in the long-term because obesity prevalence continues to grow despite an ever-increasing number of weight loss attempts. Moreover, despite documented improvements in cardiometabolic risk factors with weight loss, clear evidence for a survival benefit associated with intentional weight loss in patients with T2D is lacking. This may be due in large part to higher mortality risk associated with weight cycling in this population. Body weight fluctuation can lead to CVD risk factor variability, and both are associated with adverse cardiovascular outcomes. Higher all-cause mortality and CVD morbidity associated with weight cycling in patients with T2D was consistent across all studies, especially CVD morbidity. Further complicating the issue is the consistent finding of lower mortality risk associated with higher BMI in individuals with T2D. These observations present a conundrum for treatment and prevention strategies that focus primarily on weight loss.

Exercise training interventions typically improve cardiometabolic risk factors by a magnitude comparable to that shown in weight-loss interventions. Even more importantly, PA and CRF are consistently and strongly associated with lower T2D incidence and mortality among those with obesity and T2D. Thus, focusing on increasing PA and improving fitness (both cardiorespiratory and muscular) might be a more straightforward approach to prevention and treatment of T2D. Because dose-response studies show that the steepest risk reductions for T2D are observed when sedentary, low-fit individuals become more active, even modest increases in PA and fitness could have considerable impact in reducing morbidity and mortality risk. A focus on increasing PA and improving fitness, without a specific weight loss target, may also help to minimize weight cycling associated sequelae.
